# Effects of Acute Aerobic Exercise on Response Inhibition in Adult Patients with ADHD

**DOI:** 10.1038/s41598-019-56332-y

**Published:** 2019-12-27

**Authors:** A. Mehren, J. Özyurt, C. M. Thiel, M. Brandes, A. P. Lam, A. Philipsen

**Affiliations:** 1 Biological Psychology Lab, Department of Psychology, School of Medicine and Health Sciences, Carl von Ossietzky Universität Oldenburg, Oldenburg, Germany; 2 Psychiatry and Psychotherapy, School of Medicine and Health Sciences, University Hospital Karl-Jaspers-Klinik, Carl von Ossietzky Universität Oldenburg, Oldenburg, Germany; 3 Research Center Neurosensory Science, Carl von Ossietzky Universität Oldenburg, Oldenburg, Germany; 4 Cluster of Excellence “Hearing4all”, Carl von Ossietzky Universität Oldenburg, Oldenburg, Germany; 5 Leibniz Institute for Prevention Research and Epidemiology - BIPS GmbH, Department of Prevention and Evaluation, Unit Applied Health Intervention Research, Bremen, Germany; 6 Department of Psychiatry and Psychotherapy, University of Bonn, Bonn, Germany

**Keywords:** Human behaviour, ADHD

## Abstract

Previous studies suggest beneficial effects of aerobic exercise on executive functions, which are a core deficit in ADHD. The aim of the present fMRI study was to investigate acute effects of aerobic exercise on inhibitory control and related brain activation in adult patients with ADHD. 23 patients and 23 matched healthy controls performed on a Go/No-go task in an MRI scanner, following both, an exercise condition involving 30 min of cycling at moderate intensity, and a control condition. ADHD patients compared to healthy controls showed increased brain activation during successful inhibition in the exercise compared to the control condition in parietal, temporal, and occipital regions. Exercise did not improve behavioral performance in either group, but in ADHD patients, exercise-related increases in brain activation and behavioral task performance (i.e., correct inhibition rate) negatively correlated with correct inhibition rate in the control condition. Thus, patients with worse inhibition performance showed stronger exercise-related enhancements, indicating that the lack of improvements on the behavioral level for the whole patient group could be due to ceiling effects. Our findings might be an important step in understanding the neural basis of exercise effects and could, in the long term, help in developing alternative treatment approaches for ADHD.

## Introduction

Attention deficit hyperactivity disorder (ADHD) is the most common neurobehavioral disorder in children and adolescents and often persists into adulthood^1^. Besides the core symptoms of inattention, impulsivity, and hyperactivity, many patients show deficits in executive functioning. Response inhibition, i.e., the ability to inhibit prepotent responses in order to stop or to select a more appropriate one, is one major component of executive functioning^2^. It is essential for adaptive behavior and plays an important role in the successful completion of many everyday tasks^3^. Reduced inhibitory control is viewed as a central deficit in ADHD^4^. Numerous studies have demonstrated that patients compared to healthy controls perform worse and display alterations in brain activation during tasks of response inhibition (e.g., Go/No-go task, Stop Signal task; e.g.^5,6^). The most prominent activation differences have been found in fronto-striatal and parietal regions, but alterations in activation of temporal, occipital, and subcortical structures have also been reported^7–11^.

Current treatment guidelines for ADHD recommend a multimodal approach, comprising psychopharmacological as well as psychotherapeutic and psychoeducational interventions^12–14^. However, in light of a considerable proportion of non-responders to medication^15^, several side-effects, as well as personal preferences^16^, there is a strong need to develop alternative treatment approaches^17,18^. Physical exercise has been shown to improve cognitive functions in healthy and clinical populations (for reviews see^19–21^). Since functions frequently impaired in ADHD seem to benefit the most from physical exercise, exercise may be highly relevant for the treatment of ADHD. Several studies have already demonstrated that a single session of aerobic exercise (i.e., acute exercise) can lead to immediate improvements in executive functions in children with ADHD^22–27^ (for review see^28^). In adults with ADHD, far fewer studies have examined the acute effects of exercise and results are less clear. While Gapin *et al*.^29^ found improved inhibitory control in college students with ADHD post compared to pre exercise, Fritz and O’Connor^30^ observed exercise-related enhancements in motivation and mood in a group of adult male patients, but no effects of exercise on attentional performance or hyperactivity. However, in a recent study from our lab, adults with ADHD showed improved reaction times during a flanker task following 30 min of exercising compared to a control condition, which we interpreted as enhancements in attentional processes and speed of processing due to acute exercise^31^.

Neurophysiological mechanisms that may account for the beneficial effects of acute exercise on cognition include elevated arousal, catecholamine concentrations^32–34^, brain derived neurotrophic factor (BDNF)^35,36^, and cerebral blood flow (CBF)^37–39^. Interestingly, these mechanisms overlap with those implicated in the pathology of ADHD, strengthening the assumption that patients with ADHD might particularly benefit from exercise. In addition, the suggested mechanisms are closely related to functioning of the prefrontal cortex, which could underlie the beneficial effects of exercise on executive functions^20,40–43^. While direct assessments of exercise-induced changes in central processes have been conducted only in animal studies (see e.g.^44–46^), the number of studies including electrophysiological measures to assess the neural effects of exercise in human participants is rapidly increasing. In children with ADHD, EEG studies have related acute exercise-induced improvements in cognition to changes in different components of the event-related potential^23,26,27^.

On the other hand, still very few studies have included fMRI to investigate the immediate changes in neuronal activity during executive function tasks due to exercise. In healthy children and young adults respectively, two fMRI studies revealed exercise-specific changes in activation of the parietal cortices and hippocampus^47^, as well as frontal areas^48^ during a working memory task. In addition, MacIntosh *et al*.^49^ observed decreases in activation of the parietal operculum during inhibitory performance as measured by a Go/No-go task following acute exercise compared to a pre-testing in healthy adults. A recent study from our lab focused on exercise intensity effects on executive task performance in healthy adults. We observed increased brain activation during a Go/No-go task in fronto-parietal regions following acute moderate-intensity exercise compared to a control condition, whereas high-intensity exercise decreased activation in those regions^50^. There were however no significant effects of acute exercise on brain activation patterns during a flanker task. Notably, we were also not able to show exercise-induced modifications in brain activity during flanker task performance in adults with ADHD, although patients improved in behavioral task performance^31^. This could imply that specific aspects of executive functioning might benefit more from exercise than others. The flanker and Go/No-go tasks are both measures of inhibitory control and attention, but measure slightly different components. While the main aspect assessed in the flanker task is interference control at stimulus level, which requires suppressing distracting stimuli and competing response tendencies, the Go/No-go task primarily measures inhibition at the response level, involving the control of motor responses^51–53^.

The present study therefore aimed at investigating the effects of a single session of aerobic exercise on response inhibition as assessed by a Go/No-go task and associated changes in task-related brain activation in adult patients with ADHD. Patients and healthy controls participated in both, a 30 min exercise condition, where they cycled on a stationary ergometer with moderate intensity, and an active control condition. Following both conditions, they performed on the Go/No-go task while functional MR images were acquired. We hypothesized that acute exercise would enhance response inhibition performance and task-related brain activation in the exercise compared to the control condition in both, adults with ADHD and in the healthy control group. However, as previous studies have observed larger effects of exercise in individuals showing poorer cognitive functioning^19,43,54^, we expected greater enhancements in patients than in healthy controls.

## Material and Methods

### Participants

This study was conducted within a larger project, which focused on effects of acute exercise on cognitive performance in a sample of patients with ADHD and healthy controls^31,50^. For the current sample, we recruited 46 adult participants (23 patients with ADHD and 23 healthy controls). Participant recruitment was conducted as described in Mehren *et al*.^31^: ADHD patients were recruited through the outpatient clinic of the Department of Psychiatry and Psychotherapy at the University of Oldenburg. All patients had received the diagnosis of ADHD according to international guidelines^13,14^, based on the diagnostic criteria of the Diagnostic and Statistical Manual of Mental Disorders (4th ed.; DSM-IV; American Psychiatric Association, 1994)^55^. Patients were diagnosed by a specialist psychiatrist following a detailed clinical and psychosocial interview that integrates somatic differential diagnosis, the patients’ psychiatric and developmental history, and observer reports. To further assess ADHD symptoms and general psychopathology, following questionnaires were obtained: the German versions of the ADHD Self Rating Scale (ADHS-SB)^56^, the Wender Utah Rating Scale (WURS-k)^57^, the Conners’ Adult ADHD Rating Scale–Self-Report: Long Form (CAARS-S:L)^58^, and the Symptom-Checklist-90 (SCL-90-R)^59^. Healthy controls were recruited via announcements in the internet and were age- and gender-matched to the patients.

The following exclusion criteria were applied for all participants: i) neurological disorders, ii) severe psychiatric disorders, which were assessed using the German versions of the Structured Clinical Interview for DSM-IV (SCID-I)^60^, the SCID-II screening questionnaire for personality disorders^61^ and the Beck Depression Inventory (BDI-II)^62^, iii) autism spectrum disorders, iv) psychotropic drugs (for patients: psychotropic drugs different from medication for ADHD), v) contraindications for MRI, and vi) health conditions which could interfere with cycling tasks. To further evaluate health conditions, participants completed an electrocardiogram and a health questionnaire before participation. One patient was excluded from the analysis due to technical problems during data acquisition (failure of the keypad to record responses), and two patients were excluded due to strong head movements during MRI, so that 20 patients and their respective 20 matched healthy controls were included in the final analyses. Their clinical and demographic characteristics are reported in Table 1.

**Table 1:** Demographic and clinical characteristics among patients with ADHD and healthy controls.

Variable	ADHD mean ± SD	Controls mean ± SD	t-statistic^1^	p-value
Age (years)	31.4 ± 9.6	29.5 ± 7.0	0.69	0.49
BMI (kg/m²)	25.6 ± 4.3	24.1 ± 2.6	1.28	0.21
HR_max_ (beats/min)	179.7 ± 9.4	187.2 ± 10.1	−2.43	0.020^*^
VO_2peak_ (mL/min/kg)	36.6 ± 7.5	42.0 ± 7.3	−2.30	0.027^*^
VO_2peak_ (% ranking)	40.4 ± 22.4	52.8 ± 21.7	−1.77	0.08
BDI	10.1 ± 6.6	2.2 ± 2.8	4.90	<0.001^*^
ADHS-SB	30.9 ± 9.0	4.8 ± 4.4	11.62	<0.001^*^
WURS-k	42.9 ± 15.4	8.4 ± 6.8	9.20	<0.001^*^
PA total score	8707 ± 8964	4114 ± 3462	2.14	0.043^*^
PA work	4699 ± 8198	383 ± 898	2.34	0.030^*^
PA transportation	1494 ± 2499	938 ± 877	0.94	0.35
PA domestic	912 ± 1067	1000 ± 2427	−0.15	0.88
PA leisure	1607 ± 1795	1793 ± 1854	−0.32	0.75
PA walking	1968 ± 2254	778 ± 1060	2.14	0.042^*^
PA moderate	3335 ± 3391	2066 ± 2541	1.34	0.19
PA vigorous	3404 ± 5989	1270 ± 1854	1.52	0.14
Stimulant medication^a^	n = 3	—	—	—

*p < 0.05; ^1^two-sample t-test; *df* = 38. ADHD: N = 20 (3 females); Controls: N = 20 (4 females); SD = standard deviation; ^a^current medication, discontinued 48 hours prior to each visit. BMI = body mass index; HR_max_ = maximal heart rate as assessed by maximal exercise test; VO_2peak_ (% ranking) = peak oxygen uptake (mL/min/kg) as tested by maximal exercise test transformed into age- and gender-adapted percentiles of healthy people; BDI = Beck Depression Inventory; ADHS-SB = ADHD Self Rating Scale; WURS-k = Wender Utah Rating Scale, retrospective assessment of childhood ADHD; PA = physical activity as assessed by the International Physical Activity Questionnaire, expressed in MET-minutes/week.

The study was conducted in accordance with the Declaration of Helsinki^63^ and all procedures were approved by the ethics committee of the University of Oldenburg. Written informed consent was obtained from all participants prior to study participation.

### Experimental task

The experimental paradigm was programmed using Cogent 2000 v125 (http://www.vislab.ucl.ac.uk/cogent.php) and Matlab R2015b (The MathWorks, Inc.) and was projected onto a screen and presented to the participants in the scanner through a mirror on the head coil. The distance from the eyes of the participants to the screen was 50 cm. Stimuli were white and displayed on a black background. An MR-compatible keypad (NAtA Technologies, Coquitlam, Canada) was used to respond with the right hand (see also^31,50^).

To assess the participants’ ability to inhibit a prepotent response and to sustain attention, a Go/No-go task as described in Mehren *et al*.^50^ was utilized. Letters of the alphabet were presented in an event-related design. Each trial started with the presentation of a single letter for 0.25 sec, followed by a variable post stimulus interval during which a fixation cross was visible. Trial duration was either 2, 6, or 8 sec, with predominantly shorter trials (mean trial length = 3.5 sec). Participants were instructed to respond to every letter (Go trials) by pressing a key with their right index finger, except to the letter ‘X’ (No-go trials). They were told to focus on the fixation cross during the entire experiment and to respond as fast and accurate as possible. The task took 12 min, with 200 trials in total being presented. The proportion of No-go trials (‘X’) was 0.35. The sequence of letters and the presentation of No-go trials were randomized.

### Questionnaires

To assess participants’ subjective amount of physical activity, the German long form of the International Physical Activity Questionnaire (IPAQ-LF; www.ipaq.ki.se) was completed. The IPAQ is a self-report questionnaire that comprises 27 items asking for the time (number of days and minutes/day) spent on walking, moderate, and vigorous physical activity during the last seven days in four domains of daily life: work, transportation, domestic chores and gardening, and leisure-time. For scoring, we used the guidelines provided on the IPAQ website. For each domain and each intensity level of physical activity, MET-minutes (Metabolic Equivalent of Task; minutes weighted by energy costs of the activity) were computed. In the end, an overall physical activity score was calculated (see also^31,50^).

### Experimental procedure

The experimental procedure was the same as described in Mehren *et al*.^31^: Subjects participated in three sessions which were separated by a minimum time interval of two days to prevent aftereffects of exercising on subsequent sessions. During a pre-experimental session, they completed a maximal exercise test, during the following two experimental sessions, they participated in the exercise and the control condition. The sequence of the conditions was counterbalanced among participants, but each patient and his/her respective matched healthy control performed the same sequence of conditions. Participants were asked to refrain from any physical exercise on the test days. If on ADHD medication, patients (n = 3) had to pause medication at least 48 hours prior to each visit.

#### Maximal exercise test

To determine participants’ individual maximal heart rate (HR_max_) and to evaluate their cardiorespiratory fitness, they completed a maximal exercise test on a stationary bicycle. They started cycling at 90 W and each 3 min, the resistance was increased by 40 W until the participant was subjectively exhausted (unable to continue pedaling). The participants’ heart rate and oxygen consumption were recorded continuously by way of a chest strap heart rate monitor (Polar RCX5, Polar Electro Oy, Finland) and a spirometer (Oxycon Mobile, CareFusion, Heidelberg, Germany), respectively. At the end of this test, HR_max_ and peak oxygen consumption (VO_2peak_ in mL/min/kg) were registered. Cardiorespiratory fitness was determined by transforming VO_2peak_ into age- and gender-adapted percentiles (VO_2peak_ % ranking) using normative data of the American College of Sports Medicine (ACSM)^64^.

#### Experimental sessions

To minimize practice effects on the experimental tasks^65^, we decided for a repeated measures design with a control condition on a different day without pre-intervention measures. The first experimental session started with a practice trial of the experimental tasks inside the scanner to familiarize with MR environment and to ensure correct task performance. Depending on the sequence of conditions, subjects then participated either in the exercise or control condition. In the exercise condition, they continuously cycled on an ergometer for 30 min. During the whole routine, target HR was between 50 and 70% of the individual HR_max_. Following 5 min of warm-up, mean %HR_max_ was 66.4 (SD = 3.7, 95% CI [65.1, 67.7]), which is in the range of moderate intensity^64^. The heart rate was continuously recorded using a chest strap heart rate monitor (Polar RCX5; Polar Electro Oy, Finland) and monitored by the experimenter.

In the control condition, participants watched the Movie for the Assessment of Social Cognition (MASC-MCk)^66^, which is similar to a soap opera. Short sequences about four young people meeting for a dinner are presented and participants have to answer questions related to the actors’ mental states. They are told that answers are subjective and that there are no incorrect answers. The MASC was chosen to keep participants engaged in a task which is entertaining but not cognitively demanding. Completion of the MASC took approximately 30 min. Notably, performance in the MASC is very easy and did not differ between patients and healthy controls. After each condition, participants entered the MR scanner and followed the same procedures. At first, they performed on a visual task and a flanker task, which took around 15 minutes to complete and whose results are reported elsewhere^31^. Then they performed on the Go/No-go task, which we focus on in the present study. At the end of the session, the visual task was presented again (see^31^) and a structural scan was obtained.

### fMRI data acquisition

Imaging was conducted on two 3-Tesla MRI Scanners (Siemens MAGNETOM Verio, 12-channel head array and Siemens Magnetom Prisma, 64-channel head array; Siemens AG, Erlangen, Germany). For each patient and his/her respective matched healthy control, imaging data was acquired in the same scanner. During performance on the Go/No-go task, functional images with BOLD-contrast were obtained (390 multislice T2*-weighted gradient echo planar imaging (EPI) volumes, time of repetition (TR) = 1750 ms, time of echo (TE) = 30 ms, flip angle (FA) = 80°, Field of View (FoV) = 200 × 200 mm², voxel size = 3.0 mm³, matrix size: 64 × 64, 31 slices, recorded sequentially in an ascending order with a 1 mm gap). Each volume covered the whole brain, except of the lowermost part of the cerebellum. T1-weighted structural images were acquired using magnetization prepared rapid gradient-echo (MPRAGE) sequence (1 mm³ isotropic voxels, 176 slices, FoV = 250 × 250 mm, TR = 1900 ms, TE = 2.52 ms, FA = 90°).

### Data analysis

#### Behavioral analysis

Behavioral data analyses were performed using SPSS Statistics 22 (IBM, Armonk, NY, USA) and RStudio 3.2.2^67^. Four types of Go/No-go task trials were classified: hits (correct responses to non-X letters) and omissions (omitted responses to non-X letters), as indicators of the ability to sustain attention over time; and correct inhibitions (successful inhibitions of responses to the X letter) and false alarms (failed inhibitions to the X letter), as indicators of the ability to inhibit a prepotent response. The proportions of hits (hit rate = number_hits_/number_Go trials_) and correct inhibitions (correct inhibition rate = number_correct inhibitions_/number_No-go trials_) were calculated as measures of accuracy. Based on the signal detection theory^68^, the sensitivity index d’ was computed (d’ = Z(hit rate) – Z(false alarm rate)). This is a bias-free measure of the subject’s ability to discriminate targets (signal) from distractors (noise). In addition, mean reaction times and reaction time variabilities for hits were calculated (see also^50^).

To examine effects of group and condition, a 2 (group: ADHD vs. Healthy controls) X 2 (condition: Exercise vs. Movie) mixed factorial repeated measures ANOVA was calculated. Paired t-tests and two-sample t-tests were used to investigate condition-specific and group differences. Our primary interest was in the effects of exercise on response inhibition. Therefore, we determined the sensitivity index d’ as well as the correct inhibition rate as primary outcome measures. As response inhibition is difficult to separate from attentional processes^69^ and exercise might influence multiple aspects of executive functioning, we also defined the hit rate as a primary outcome measure. For those three measures, we applied Bonferroni correction to account for multiple comparisons (significance level of p ≤ 0.017, two-sided). To account for group differences in cardiorespiratory fitness levels (see Table 1) and based on previous studies showing that fitness might modulate the effects of acute exercise on cognition (e.g.^31,50^), we performed the main analysis (mixed factorial repeated measures ANOVA) again, including VO_2peak_ values as covariate. To investigate associations between exercise effects and participants’ cognitive performance levels, we calculated Pearson correlations between exercise-related changes in behavioral measures (differences between exercise and control condition) and performance in the control condition for each group separately. For those exploratory analyses, we set the significance level to p ≤ 0.05 (two-sided) and performed no correction for multiple testing.

#### fMRI analysis

MRI data analysis was conducted in SPM12 (Wellcome Trust Centre for Neuroimaging, London, UK) based on Matlab R2016a. The following preprocessing steps were performed: i) realignment of the functional images to the mean image volume, ii) coregistration of the functional and anatomical T1-weighted images, iii) segmentation of the anatomical image, iv) spatial normalization to the MNI stereotactic space (Montreal Neurological Institute, Quebec, Canada), and v) spatial smoothing (full-width-half-maximum = 8 mm). We inspected motion parameters of each participant visually and computed maximal total and maximal scan-to-scan displacement. Two patients showed excessive head motion (exceeding 2 mm of scan-to-scan displacement or 4 mm of total displacement) and were accordingly excluded from further analysis. For the remaining patients (n = 20) and their respective matched healthy controls, we calculated two-sample t-tests to investigate whether maximal translational and rotational head displacements differed between groups. Differences in head motion between conditions were examined using paired t-tests.

On single-subject level, statistical analyses were conducted based on the standard general linear model (GLM) in SPM12. For the Go/No-go task, we performed event-related analyses. Stick functions that were time-locked to the event onsets modeled task-related signal increases. The design matrix consisted of ten regressors: four regressors of interest (hits, correct inhibitions, false alarms, and omissions) and the six motion regressors as nuisance regressors. Due to the potential effects of acute exercise on different aspects of executive functioning and due to a proposed overlap of neural circuits for processes of inhibition, attention, and response selection^3,69,70^, we defined the three contrasts *hits*, *correct inhibitions*, and *correct inhibitions – hits* as primary outcome measures. In order to eliminate non-physiological low-frequency noise, a high-pass filter (1/128 Hz) was administered. An AR(1) model corrected for temporal autocorrelation across the fMRI time series and the standard SPM approach of Restricted Maximum Likelihood was utilized to estimate GLM parameters. For the purpose of group analyses, we conducted two-sample t-tests with the differential contrasts of *Exercise – Movie* and *Movie – Exercise* in order to examine possible interactions between group and condition. To account for differences in cardiorespiratory fitness between patients and controls, we performed an additional analysis, including VO_2peak_ values as covariate. To investigate differences between groups for each condition separately, two-sample t-tests for *Movie* and *Exercise* comparing patients to healthy controls were performed. To compare the two conditions within groups, we conducted paired t-tests. To test for associations between changes in brain activation due to exercise and behavioral performance level, we calculated correlations between differences in brain activation between the two conditions and behavioral performance in the control condition for each group separately. We applied an initial voxel threshold of 0.001 uncorrected with no restrictions to cluster size and accounted for multiple testing on cluster level (corrected pFWE < 0.05). As there is only scarce knowledge on the acute effects of exercise on brain activation, with heterogeneous results obtained in the few available fMRI studies, we decided not to restrict our analyses to a priori defined regions of interest and instead performed whole-brain analyses. Stereotaxic coordinates are reported in MNI space and figures are displayed according to neurological convention.

## Results

### Demographic data

Participants’ demographic and clinical data and test scores are reported in Table 1. Patients and healthy controls did not differ in age, gender, and BMI, but patients showed higher scores for ADHD symptoms and depression than healthy controls. In addition, patients scored higher in the International Physical Activity Questionnaire for the domains walking and physical activity at work, resulting in a higher total score. However, patients showed lower cardiorespiratory fitness levels as indicated by VO_2peak_ compared to healthy controls.

### Timing of task administration

The average time interval between the end of exercising and the beginning of the Go/No-go task was 20.9 min (SD = 1.8, range 19–27).

### Behavioral results

Group means for behavioral measures are summarized in Table 2. For our primary outcome measures, there were neither interaction effects between group and condition nor main effects of group or condition. Including cardiorespiratory fitness (VO_2peak_) as covariate did not reveal different outcomes.

**Table 2:** Behavioral performance during the Go/No-go task for each group and condition.

Variable	ADHD (n = 20)		Controls (n = 20)	
	Movie mean (SE)	Exercise mean (SE)	Movie mean (SE)	Exercise mean (SE)
Sensitivity index (d’)	3.32 (0.13)	3.36 (0.18)	3.30 (0.13)	3.41 (0.17)
Hit rate	0.982 (0.005)	0.971 (0.014)	0.965 (0.014)	0.964 (0.011)
Correct inhibition rate	0.883 (0.025)	0.901 (0.018)	0.908 (0.020)	0.920 (0.025)
RT hits (ms)	524 (11)	523 (11)	514 (13)	521 (15)
RTV hits (ms)	110 (6)	104 (8)	103 (5)	112 (6)

RT = reaction time; RTV = reaction time variability; SE = standard error of the mean.

### Correlation of behavioral performance measures

In patients, correct inhibition rate in the control condition negatively correlated with the difference in correct inhibition rate between the two conditions (Exercise – Movie; r² = 0.49, p = 0.001). Patients with worse performance in the control condition showed greater exercise-related enhancements. Note that one patient showed strong impairments in task performance (correct inhibition rate of 0.49 in the control condition and 0.61 in the exercise condition), although correct understanding of the task was ensured during task instructions and the practice trial. Please note that this patient responded to 95% of Go trials correctly, which is well in the range of the other patients included in this study (range of 91–100% of correct Go trials) and clearly shows that he did not perform on chance level. However, to account for this extreme data point, we decided to perform an additional, more robust correlational analysis using Spearman’s rank order correlation and received very similar results to the original analysis (r² = 0.68, p < 0.001). In the healthy control group, there was no significant correlation between exercise-related behavioral performance changes and performance in the control condition.

### Head motion

There were no significant differences in head motion during fMRI (maximal scan-to-scan and total displacement for translation and rotation parameters) between patients with ADHD and healthy controls. In both groups, motion parameters did also not differ between the exercise and the control condition.

### fMRI results

Our primary interest was related to differences in brain activation between the exercise and the control condition. However, to test whether our task revealed brain activation patterns similar to those reported in the previous literature, we first analyzed brain activation during correct No-go trials (contrast *correct inhibitions*) for each of the conditions in healthy controls and patients separately (see Supplement 1). In the control condition (Movie), patients showed significant activation in occipital, temporal, and cerebellar regions, whereas healthy controls showed activation in large clusters located in frontal, parietal, temporal, occipital, cerebellar, and subcortical regions. These clusters included areas consistently reported to be activated during tasks of response inhibition and attention in previous studies (e.g., inferior frontal, insula, anterior cingulate, sensorimotor areas)^70–73^. Comparing brain activation during *correct inhibitions* in the control condition between patients and healthy controls did, however, not yield any significant results. In the exercise condition, both, patients and healthy controls, showed brain activation in frontal, parietal, temporal, occipital, and cerebellar regions, including those known to be relevant for response inhibition (e.g., inferior frontal, insula, sensorimotor areas). Again activations were not significantly different between groups.

**Figure 1:**
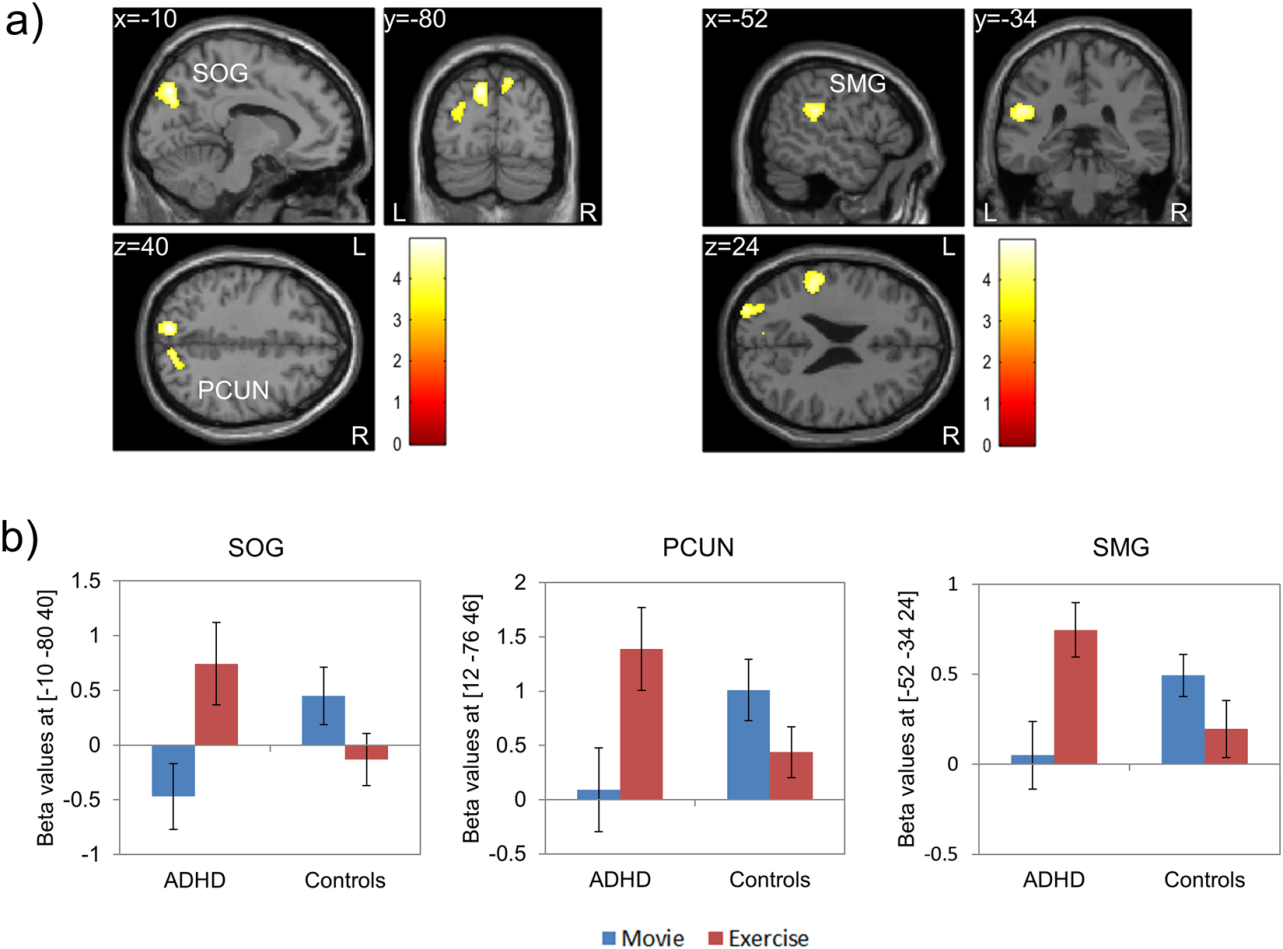
Brain activation during the Go/No-go task for the contrast *correct inhibitions*: (**a**) activation specific to *ADHD (Exercise – Movie) – Controls (Exercise – Movie)*; (**b**) mean beta values of peak coordinates with standard error for each group and condition separately. Activation differences were found in three clusters with peak activation in the left superior occipital gyrus (SOG), right precuneus (PCUN), and left supramarginal gyrus (SMG). p < 0.05 (FWE-corrected on cluster level, initial voxel threshold 0.001 uncorrected).

Regarding the different effects of exercise on brain activation in ADHD and healthy controls, we observed a significant interaction between the factors group and condition for *correct inhibitions* in three clusters (Fig. 1a): the first cluster extended from the left superior occipital gyrus to superior parietal and middle occipital regions; the second cluster presented with a peak activation in the right precuneus and extended to superior occipital and superior parietal regions; the third cluster had its peak in the left supramarginal gyrus and extended to the superior temporal gyrus and the rolandic operculum. BOLD responses within those clusters increased in the ADHD patient group and decreased in the healthy control group in the exercise compared to the control condition (Movie; Fig. 1b). Peak coordinates and statistical results are given in Table 3. In patients, exercise-related increase in brain activation in the cluster comprising the left supramarginal gyrus, superior temporal gyrus, and the rolandic operculum (difference in peak beta values between the exercise and control condition) positively correlated with exercise-related enhancements in behavioral performance (difference in correct inhibition rate between exercise and control condition; Pearson correlation: r² = 0.288, p = 0.015; Spearman’s rank order correlation: r² = 0.26, p = 0.021). In other words, patients with stronger exercise-related increases in brain activation also showed stronger enhancements on behavioral level. In the healthy control group, exercise-related decrease in brain activation in this cluster was not correlated with exercise-related changes in behavioral performance. Including cardiorespiratory fitness (VO_2peak_) as covariate in the interaction analysis revealed significant activations in the same clusters as in the original model (except for the cluster peaking in the left supramarginal gyrus, which remained only marginally significant (p = 0.051)).

**Table 3:** Brain activation during *correct inhibition* trials of the Go/No-go task.

Group, Condition	Region of peak activation	MNI coordinates (x, y, z)	Cluster size	t-statistic	z-statistic	p^*^
ADHD (Exercise – Movie) – Controls (Exercise – Movie)^1^	L superior occipital	−10, −80, 40	662	4.94	4.31	0.001
	R precuneus	12, −76, 46	359	4.71	4.15	0.014
	L supramarginal	−52, −34, 24	315	4.63	4.10	0.023
ADHD (Exercise - Movie)^2^	L middle occipital	−36, −90, 10	1433	6.03	4.45	<0.001
	R middle occipital	40, −76, 16	1326	5.35	4.13	<0.001
	R supramarginal	36, −38, 42	276	5.22	4.06	0.037
	L inferior parietal	−28, −46, 50	517	4.74	3.81	0.003
	L inferior parietal	−52, −22, 38	378	4.42	3.62	0.012
Controls (Movie – Exercise)^2^	not significant					

^*^FWE-corrected on cluster level (initial voxel threshold 0.001 uncorrected); ^1^two-sample t-test; ^2^paired t-test.

Additional paired t-tests showed that in patients, brain activation during *correct inhibitions* increased in the exercise compared to the control condition in five clusters (Table 3): the first cluster was located in the left middle, inferior, and superior occipital gyri, and extended to the middle temporal gyrus; the second cluster presented with peak activation in the right middle occipital gyrus and comprised the superior parietal gyrus, the cuneus, and superior occipital and middle temporal regions; the third cluster extended from the right supramarginal gyrus to the superior parietal and postcentral gyri; the fourth cluster was located in the left inferior parietal gyrus, extending to the superior parietal gyrus, precuneus, and postcentral gyrus; the fifth cluster extended from the left inferior parietal gyrus to the superior temporal gyrus, supramarginal gyrus, postcentral gyrus, and the rolandic operculum. For the healthy control group, paired t-tests revealed no significant difference in brain activation between the two conditions.

No other contrasts of interest reached statistical significance neither between nor within groups.

### Correlation of brain activation and behavior

**Figure 2:**
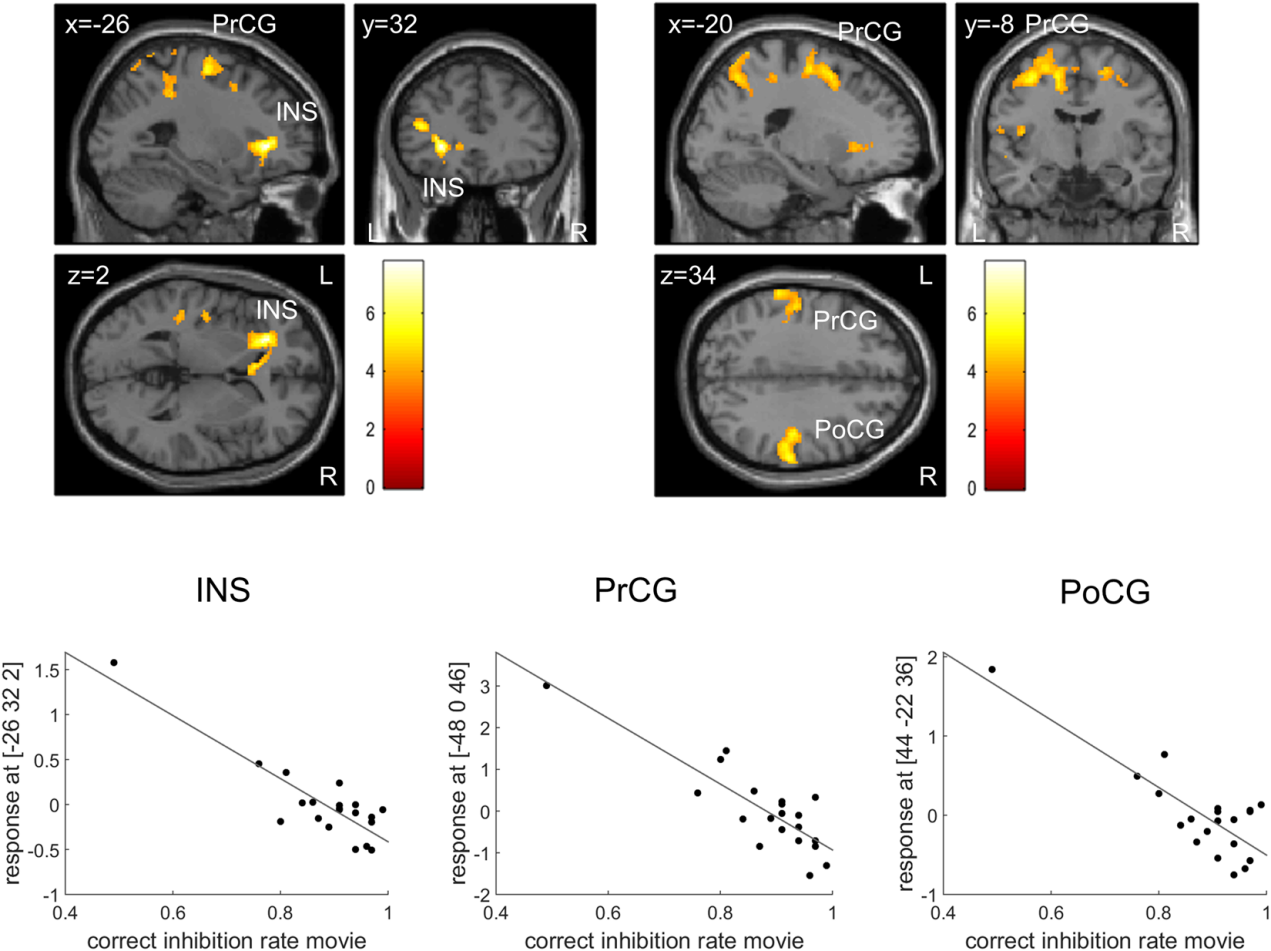
Correlation between exercise-related changes in brain activation during *correct inhibitions* and task performance in the control condition. In ADHD patients, the difference in activation between the two conditions (*Exercise – Movie*) negatively correlated with correct inhibition rate in the control condition in three clusters with peak activation in the left insula (INS), left precentral gyrus (PrCG), and right postcentral gyrus (PoCG). p < 0.05 (FWE-corrected on cluster level, initial voxel threshold 0.001 uncorrected).

In patients, brain activation during *correct inhibitions* in the exercise compared to the control condition negatively correlated with behavioral performance (i.e., correct inhibition rate) in the control condition. In other words, patients with worse behavioral performance (i.e., lower correct inhibition rate) in the control condition showed stronger exercise-related increases in brain activation in three clusters (Fig. 2): the first cluster extended from the left insula to the left precentral and postcentral gyri, inferior frontal gyrus (triangular, orbital, and opercular parts), and middle frontal gyrus; the second cluster presented with peak activation in the left precentral gyrus, and extended to the right precentral and superior frontal gyri, left middle frontal gyrus, postcentral gyri of both hemispheres, supplementary motor area, precuneus, and midcingulate gyrus, left insula and supramarginal gyrus, and to left inferior parietal and middle temporal regions; the third cluster comprised the right postcentral and supramarginal gyri. Peak coordinates and statistical results are reported in Table 4. To account for the patient showing very low behavioral task performance (see Fig. 2), we conducted additional Spearman’s rank order correlational analyses between correct inhibition rate in the control condition and the differences in peak beta values between the exercise and control condition. For the two clusters peaking in the left insula and left precentral gyrus, the negative correlation between performance in the control condition and increases in brain activation due to exercise was also significant in these analyses ([−26 32 2]: r² = 0.35, p = 0.006; [−48 0 46]: r² = 0.50, p = 0.001), while we found only a trend for the peak in the right postcentral gyrus ([44–22 36]: r² = 0.17, p = 0.069). In the control group, brain activation in the exercise compared to the control condition did not correlate with behavioral performance in the control condition.

**Table 4:** Correlation between exercise-related changes (*Exercise – Movie*) in brain activation during *correct inhibition* trials of the Go/No-go task and task performance in the control condition (correct inhibition rate) in ADHD patients.

Region of peak Activation	MNI coordinates (x, y, z)	Cluster size	t-statistic	z-statistic	p^*^
L insula	−26, 32, 2	919	7.75	5.08	<0.001
L precentral	−48, 0, 46	4462	7.01	4.81	<0.001
R postcentral	44, −22, 36	391	6.25	4.50	0.007

^*^FWE-corrected on cluster level (initial voxel threshold 0.001 uncorrected).

## Discussion

The aim of the current fMRI study was to investigate acute effects of aerobic exercise on response inhibition in adult patients with ADHD. We hypothesized exercise-induced improvements in Go/No-go task performance and associated changes in brain activation in both, patients and in healthy controls, expecting patients to benefit to a greater extent. Although ADHD patients and healthy controls did not improve in behavioral task performance, patients compared to healthy controls showed exercise-related increases in brain activation during correct inhibition trials in parietal, temporal, and occipital regions. In addition, exercise-induced changes in patients’ brain activation and behavioral performance correlated with their behavioral performance in the control condition, in the sense that patients with worse performance showed greater exercise-related activation increases and enhancements on behavioral level.

### Acute exercise effects on behavioral task performance

Contrary to our hypothesis, we did not observe effects of acute exercise on behavioral performance in the Go/No-go task. Although the Go/No-go task has shown to be sensitive to exercise effects in previous studies, some studies have also failed to achieve improvements in task performance due to exercise (for review see^20^). In addition, exercise effects on cognitive processes seem to depend on several factors (e.g., characteristics of exercise, timing of task, and characteristics of participants). There is evidence that individuals with lower cognitive performance levels as well as individuals undergoing developmental changes (i.e., children and older adults) are more susceptible to benefit from exercise^19,43,54^. Previous studies on exercise effects have mainly been conducted with children with ADHD, whereas the few studies with adult patients have revealed mixed results. While Gapin *et al*.^29^ found exercise-related improvements in inhibitory control in college students with ADHD, Fritz and O’Connor^30^ failed to report effects of acute exercise on attention and hyperactivity in adult male patients. Note that Gapin *et al*.^29^ used a pre-post design instead of including a control condition, introducing the possibility of practice effects. In our previous study, we observed enhanced reaction times during a flanker task after acute exercise in adults with ADHD^31^. However, in this study, patients’ reaction times in the control condition were significantly higher than those of the healthy control group, leaving still room for improvements. In the present study, ADHD patients’ task performance was comparable to the performance of the healthy control group. Notably, both groups’ performance levels were on average very high and only few patients made a considerable amount of errors. Consequently, we assume that the lack of behavioral improvements following exercise was due to ceiling effects. In patients, we found a correlation between task performance in the control condition and changes in performance due to exercise, in the sense that patients with worse performance showed greater exercise-related enhancements. This finding further strengthens the assumption that ADHD patients with more severe impairments in executive functioning might show larger benefits from exercise, which is in line with the finding that stimulant medication effects are stronger in patients with lower cognitive performance levels^74^. In addition, exercise effects might have been present in tasks being more difficult and requiring a heavier cognitive load^48^. The Go/No-go task we used in this study would count as rather simple type of Go/No-go tasks, minimizing the contribution of other cognitive processes (e.g., working memory).

### Acute exercise effects on task-related brain activation

As hypothesized, we found acute effects of exercise on brain activation patterns during the Go/No-go task, with a significant difference in exercise-related activation changes between the two groups for successful No-go trials (correct inhibitions). In patients, exercise compared to the control condition was associated with increased brain activation in task-related areas whereas healthy controls showed a descriptive decrease in brain activation due to exercise. The increase in brain activation in patients was also significant in within-group comparisons (paired t-tests) while the decrease in activation in healthy controls was not significant.

Brain areas whose activation changed following exercise included the superior and inferior parietal gyri, postcentral and supramarginal gyri, rolandic operculum, precuneus and cuneus, superior and middle temporal regions, and the occipital cortex. The parietal and temporal cortices as well as the cuneus have shown to be involved in tasks of response inhibition in previous studies^75–79^. Parietal regions in particular have often been related to attentional processes^80–85^. The parietal cortex receives input from the visual and somatosensory systems and gives output to the frontal motor cortex. The parietal-prefrontal circuit plays a crucial role when successfully inhibiting a response^78^. In addition, the precuneus has found to be involved in directing attention in space when imagining, preparing, or executing movements, as well as in shifting attention between motor targets, and error monitoring, e.g., during a Go/No-go task^77^. The postcentral gyrus forms the primary somatosensory cortex and is involved in response execution^77^ while the rolandic operculum is part of the secondary somatosensory cortex and has a role in stimulus elaboration, attention, and motor function^86^. The occipital cortex including the cuneus is not only responsible for basic visual processes, but has also been related to attention, working memory, response execution and inhibition, and solving of complex tasks^75–78,87,88^. Taken together, activation in brain regions involved in attention, response inhibition, sensory processing, and motor performance was increased after exercise in ADHD patients. This could indicate facilitation of attentional and inhibitory control as well as sensory processes. For one cluster, comprising the supramarginal gyrus, superior temporal gyrus, and the rolandic operculum, the increase in brain activation was positively correlated with exercise-related enhancements on behavioral level, which may be interpreted as support for the assumption that an increase in brain activation was related to improved cognitive functioning. Even though we found no behavioral improvements for the whole patient group, which was possibly due to ceiling effects, cognitive performance after exercise might have been less effortful.

Previous studies have shown functional and structural abnormalities in various brain areas in children and adults with ADHD, including the regions described above (for reviews see^89,90^). The majority of functional imaging studies have observed hypoactivity in those regions in executive functioning tasks in patients compared to healthy controls. In our study, patients also showed no significant brain activation in areas robustly reported to be activated during response inhibition tasks (i.e., fronto-parietal activation) in the control condition, whereas healthy controls showed brain activation patterns comparable to those described for healthy subjects in the previous literature (e.g., inferior frontal gyrus, insula, parietal regions)^70–73^, which might suggest abnormalities in brain functioning in our patient sample. However, we did not find statistically significant differences in brain activation in the control condition between patients and healthy controls.

Exercise-related brain activation changes were also not present in frontal areas, which is surprising given that the proposed neurophysiological mechanisms of exercise are mainly associated with prefrontal cortex functioning and executive function is known to rely on the interaction of fronto-parietal networks. However, the finding that ADHD patients recruit alternative functional brain networks during tasks of executive function has been reported in several studies^91^. Note that to date, evidence for exercise-induced changes in neurophysiological processes is mainly based on animal studies. Only few previous studies on humans have investigated immediate changes in neuronal activity due to exercise, revealing heterogeneous results. In adults with ADHD, we could previously not show exercise-related changes in brain activation during a flanker task. Nevertheless, in patients with higher levels of cardiorespiratory fitness, we found exercise-induced activation changes in premotor, medial frontal, and temporal areas^31^. In another study from our lab with healthy participants, we observed increased brain activation during Go/No-go task performance in fronto-parietal regions following moderate-intensity exercise, while exercise at high intensity was associated with decreased activation in the those areas^50^. This is in contrast to findings in the present study rather pointing towards decreased brain activation following exercise at moderate intensity in the healthy control group. However, reduced activation following exercise was only observed in comparison to the patient group, i.e., not significant in within-group comparisons (paired t-tests). Note that moderate exercise-related increases in fronto-parietal brain activation in our previous study were accompanied by enhancements in behavioral performance, indicating improved neural processing. In general, both increased and decreased recruitment of brain resources may be related to enhanced processing, but in the present study, healthy controls showed no exercise-related changes in behavioral performance or associations between brain activation and behavior, complicating an interpretation. As baseline performance levels of healthy participants were similar high in both studies, we speculate that the difference in findings might be based on differences in sample sizes (i.e., n = 20 per group in the present study, n = 30 per group in the previous study). Notably, the few prior fMRI studies on acute exercise effects in healthy participants have also not revealed unitary results concerning the direction of activation changes. During Go/No-go task performance, MacIntosh *et al*.^49^ found exercise-related decreases in activation in the parietal operculum. On the other hand, Chen *et al*.^47^ observed increased activation in the superior and inferior parietal gyri, the hippocampus, and the cerebellum during a working memory task and Li *et al*.^48^ reported both, exercise-induced increases and decreases in activation in frontal, paracentral, and occipital regions during a working memory task.

In the present patient sample, exercise-induced changes in brain activation during correct inhibitions negatively correlated with behavioral performance (i.e., correct inhibition rate) in the control condition. Patients with lower performance levels showed stronger exercise-related increases in brain activation in three clusters, including the insula and inferior frontal gyrus, middle and superior frontal gyri, pre- and postcentral gyri, supplementary motor area, supramarginal gyrus, midcingulate cortex, inferior parietal and middle temporal regions, and the precuneus. This indicates that in patients with worse executive functioning performance, acute exercise had stronger effects on brain activation in regions typically involved in tasks of response inhibition (e.g., inferior frontal, insula, supplementary motor area). In combination with the association observed between performance in the control condition and exercise-induced changes in behavioral performance, this could imply that patients with more severe impairments in executive functioning could benefit to a greater extent from acute exercise, which would be in line with the finding that effects of methylphenidate are stronger in patients with lower cognitive performance levels^74^. Note that in our study, one patient showed very poor inhibition performance, while most of the other patients included performed high. However, this patient scored also high on ADHD questionnaires, indicating that he suffers from strong ADHD-related symptomatology. Previous studies have observed similar ranges of performance levels in ADHD patients, but with a higher rate of low performers (e.g.^92,93^). As we have reasonable grounds to assume that the patient was able to perform the task according to the instructions provided and as patients with lower performance levels reflect the typical ADHD-related deficits, we believe it is important to not exclude these data post hoc from our analyses. Instead, we performed additional analyses that are more robust to outliers. Nevertheless, it is important to note that validity of the correlations observed is limited by the distribution of the data. To systematically investigate the association between exercise effects and baseline performance, future studies should aim at recruiting patients from the full spectrum of symptom severities.

## Conclusions

The present study demonstrates effects of acute exercise on brain activation in adult patients with ADHD, which might be important for understanding the neural basis of exercise effects on executive function. As short-term effects of acute exercise are likely to accumulate to long-term changes in brain plasticity through exercise interventions, our results may be also relevant for developing alternative or add-on treatment approaches for ADHD. However, in our sample, exercise had no influence on behavioral task performance, which could be attributed to individual patient characteristics such as an already high performance level. Nevertheless, previous studies have demonstrated that changes in brain plasticity due to exercise can lead to improvements in cognitive function^94–96^. Thus, investigating exercise effects in patients showing more severe impairments as well as long-term effects of exercise in ADHD could be a topic for future investigations.

## Supplementary information

Supplementary Material

## Data Availability

The datasets generated and analysed during the current study are available from the corresponding author on reasonable request.
