# Supplementary material for: Effects of Acute Aerobic Exercise on Response Inhibition in Adult Patients with ADHD
Source: Sci Rep. 2019 Dec 27;9:19884. doi: 10.1038/s41598-019-56332-y (PMC6934617; doi:10.1038/s41598-019-56332-y)
Supplement: Supplementary file 1 — Supplementary Material [file 41598_2019_56332_MOESM1_ESM.docx]

Supplementary Material

**Effects of Acute Aerobic Exercise on Response Inhibition in Adult Patients with ADHD**

Mehren A, Özyurt J, Thiel CM, Brandes M, Lam AP, Philipsen A

**
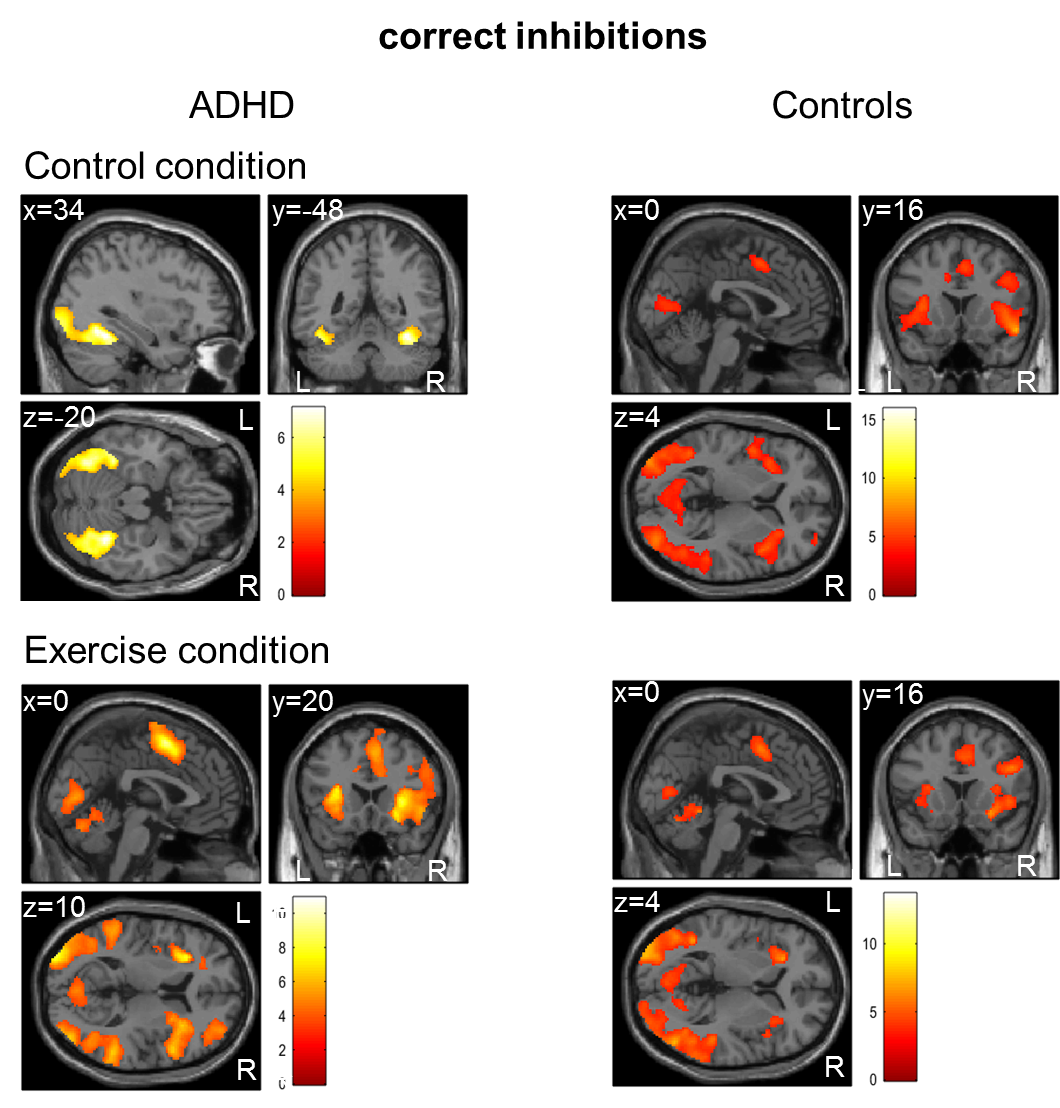
**

**Supplementary Figure 1.** Brain activation during the Go/No-go task for the contrast *correct inhibitions* in the control condition and the exercise condition for patients and healthy controls at the level p<.05 (FWE-corrected on cluster level, initial voxel threshold .001 uncorrected)

**Supplementary Table 1**

Task-related brain activation within the single conditions for correct No-go trials (contrast *correct inhibitions*) for patients and healthy controls

| **Group, Condition** | **Hemisphere(s)** | **Region of peak activation** | **Regions comprised in the cluster** | **MNI coordinates (x, y, z)** | **Cluster size** | **t-statistic** | **z-statistic** | **p*** |
| --- | --- | --- | --- | --- | --- | --- | --- | --- |
| **ADHD Movie** | R | fusiform | middle temporal  inferior + superior occipital  lingual  calcarine  cerebellum | 34, -48, -20 | 2290 | 7.12 | 4.91 | <.001 |
|  |  |  |  |  |  |  |  |  |
|  | L | inferior occipital | fusiform  middle temporal  middle occipital  cerebellum | -40, -84, -10 | 2034 | 7.00 | 4.86 | <.001 |
|  |  |  |  |  |  |  |  |  |
| **ADHD Exercise** | L + R | fusiform | inferior + superior parietal  postcentral  temporal  occipital  hippocampus  cerebellum | 34, -44, 20 | 22861 | 10.90 | 6.07 | <.001 |
|  |  |  |  |  |  |  |  |  |
|  | L + R | supplementary motor area | frontal cortex (e.g., inferior frontal, orbitofrontal, anterior cingulate, midcingulate)  pre-, post-, + paracentral  rolandic operculum  insula  pallidum  caudate nucleus  putamen | -4, 4, 56 | 12166 | 10.71 | 6.02 | <.001 |
|  |  |  |  |  |  |  |  |  |
|  | L | insula | putamen  rolandic operculum  inferior frontal  posterior orbitofrontal | -30, 24, 8 | 1024 | 8.01 | 5.23 | <.001 |
|  |  |  |  |  |  |  |  |  |
|  | L | middle frontal | inferior + superior frontal | -26, 36, 26 | 360 | 4.53 | 3.69 | 0.014 |
|  |  |  |  |  |  |  |  |  |
| **Controls Movie** | L + R | fusiform | occipital  parietal  temporal  cerebellum | -36, -58, -16 | 14138 | 15.91 | 7.04 | <.001 |
|  |  |  |  |  |  |  |  |  |
|  | R | superior temporal pole | putamen  inferior frontal  rolandic operculum  insula | 52, 14, -12 | 1231 | 8.92 | 5.53 | <.001 |
|  |  |  |  |  |  |  |  |  |
|  | L | rolandic operculum | temporal  insula  inferior frontal | -46, 2, 8 | 1166 | 6.28 | 4.57 | <.001 |
|  |  |  |  |  |  |  |  |  |
|  | R | middle frontal | inferior + superior frontal  precentral | 36, 36, 26 | 2252 | 6.18 | 4.52 | <.001 |
|  |  |  |  |  |  |  |  |  |
|  | L + R | supplementary motor area | superior frontal, medial part  anterior cingulate  midcingulate | 2, 10, 48 | 598 | 5.53 | 4.22 | <.001 |
|  |  |  |  |  |  |  |  |  |
| **Controls Exercise** | R | fusiform | temporal  hippocampus  postcentral  supramarginal  precuneus  cerebellum  occipital | 40, -58, -14 | 9425 | 13.66 | 6.66 | <.001 |
|  |  |  |  |  |  |  |  |  |
|  | L | fusiform | occipital  temporal  supramarginal  cerebellum | -38, -60, -14 | 4387 | 10.91 | 6.07 | <.001 |
|  |  |  |  |  |  |  |  |  |
|  | R | insula | putamen  inferior frontal  orbitofrontal | 28, 18, -12 | 734 | 6.85 | 4.80 | <.001 |
|  |  |  |  |  |  |  |  |  |
|  | R | inferior frontal operculum | inferior + middle frontal  precentral | 42, 10, 30 | 633 | 6.79 | 4.78 | <.001 |
|  |  |  |  |  |  |  |  |  |
|  | L | insula | putamen  inferior + middle frontal | -24, 28, 4 | 576 | 6.68 | 4.74 | <.001 |
|  |  |  |  |  |  |  |  |  |
|  | L + R | supplementary motor area | superior frontal  midcingulate | -2, 8, 46 | 852 | 6.07 | 4.47 | <.001 |
|  |  |  |  |  |  |  |  |  |
|  | L | inferior parietal | supramarginal  postcentral  superior parietal  angular | -46, -36, 44 | 1521 | 5.73 | 4.31 | <.001 |
|  |  |  |  |  |  |  |  |  |
|  | R | middle frontal | inferior frontal | 34, 36, 32 | 371 | 5.59 | 4.25 | .005 |
|  |  |  |  |  |  |  |  |  |
|  | L + R | calcarine | cuneus  lingual | -6, -72, 8 | 681 | 5.36 | 4.13 | <.001 |
|  |  |  |  |  |  |  |  |  |
|  | L | precentral | inferior + middle frontal  insula  postcentral  rolandic operculum | -48, -4, 54 | 379 | 4.92 | 3.90 | .005 |
|  |  |  |  |  |  |  |  |  |
|  | R | superior frontal | middle frontal | 24, 54, 30 | 230 | 4.35 | 3.58 | .038 |

*FWE-corrected on cluster level (initial voxel threshold .001 uncorrected)
